# Elements of Chemical Analysis, Inorganic and Organic

**Published:** 1843-04

**Authors:** 


### ART. XXII.

*Elements of Chemical Analysis, Inorganic and Organic.* By EDWARD A. PARNELL, Chemical Assistant at University College, London.—*London,* 1842. 8vo, pp. 309.

THE appearance, nearly at the same time, of three most valuable systematic treatises on Chemistry,—those of Professor Graham and Dr. Kane, and the new edition of Dr. Turner's, (edited by Dr. Gregory and Professor Liebig, and really almost a new work,) is an indication that industry and zeal in the prosecution of this science are not now confined to the continent, but that Britain is likely to take its fair share in the labour. The recent completion of the last of these works will probably induce us, at no distant period, to offer our readers such an account of them as may guide them in their choice ; and to put them in possession, as we endeavoured to do on a former occasion, of the general outlines of the progress of the science during the preceding two or three years. That we are still far behind our continental neighbours in analytic chemistry cannot be doubted ; and, independently of other causes, we think that this backwardness may be traced to the want of a good elementary treatise on the subject, which might serve as a safe and sure guide to the young chemist, when entering its difficult and perplexing path. We are not aware that any original treatise on the subject has ever been produced in England ; or that any translated works exist in our language, save Rose's Handbuch and Liebig's pamphlet on Organic Analysis. The translation of the former work is now almost out of date, being from one of its early editions ; and the work itself is much more fitted for the proficient than the learner, from the want of practical information as to the manipulations of the laboratory, and from the minuteness with which the various processes and reactions are discussed. The latter, from its limited object, is obviously not adapted to supply the deficiency.

We are quite disposed, therefore, to welcome Mr. Parnell's treatise as a valuable addition to our scientific literature, abundant as it may already seem to be ; and we feel confident that it will speedily make its way with the public. The work is divided into four chapters, or rather books : the first treating of "manipulations and reagents;" the second of the "*behaviour* of substances with reagents;" the third of "qualitative analysis ;" and the fourth of "quantitative analysis."

In the first chapter we find a general account of the *kind* of processes which the analyst has to perform, the apparatus required, and the chief precautions to be observed in the use of it; this part, which is illustrated by woodcuts, might, we think, have been extended with advantage. Next follows a section containing notices of the chief tests on reagents employed, and the means for ascertaining their purity.

The details of the second part are arranged in a tabular form, which has the advantage of exhibiting them in the state of greatest compression, and one that admits of the easiest reference. The third part consists of the application of these in qualitative analysis, with directions and examples; and it also includes an account of the use of the blowpipe, of the analysis of mineral waters, urine, and urinary calculi, and of the detection of poisons in organic mixtures. The "behaviour" of the various substances under the blowpipe is set forth in tables like those of the second part. We think some portions of this division too much condensed, and others decidedly meager. Thus the qualitative analysis of urine and the discrimination of urinary calculi are discussed in less than three pages ; and these are but an abridgment of the directions given by Berzelius, which are much behind the present state of knowledge of this subject. Thus all that is said of the detection of albumen is comprised in the following sentence: "Nitric acid gives a floccy [flocky] white precipitate with albumen, which is soluble in caustic potash after being washed, and is not reprecipitated by acetic acid." In the fourth part, on quantitative analysis, it strikes us that a very large proportion is bestowed upon mineral substances; and that the section on organic analysis is very meager. The instructions contained in an elementary work of this kind should be not only directions as to the processes to be performed, but intimations of the numerous sources of error by which the results may be disturbed, and of the mode of guarding against these. For such we look in vain in the work before us; and we would suggest to the author to extend, in the future edition which we doubt not will soon be required, those portions of the work that are of peculiar importance to the physiologist, the toxicologist, and the physician, and to make the directions for each process as simple, comprehensive, and independent as possible. We doubt not that there are many young practitioners, who would be glad to employ some of their leisure hours in such investigations, when provided with such an intelligent guide as we think Mr. Parnell might easily afford them.

As we are not chemists by profession we shall not enter into any examination of the details of this work ; the general accuracy of which is secured by the author's position at University College, and by the supervision of the illustrious Professor to whom it is inscribed.

